# Effectiveness of Using Mobile Phone Image Capture for Collecting Secondary Data: A Case Study on Immunization History Data Among Children in Remote Areas of Thailand

**DOI:** 10.2196/mhealth.4183

**Published:** 2015-07-20

**Authors:** Kasemsak Jandee, Jaranit Kaewkungwal, Amnat Khamsiriwatchara, Saranath Lawpoolsri, Waranya Wongwit, Peerawat Wansatid

**Affiliations:** ^1^ Center of Excellence for Biomedical and Public Health Informatics (BIOPHICS) Faculty of Tropical Medicine Mahidol University Bangkok Thailand; ^2^ Department of Tropical Hygiene Faculty of Tropical Medicine Mahidol University Bangkok Thailand; ^3^ Department of Social and Environmental Medicine Faculty of Tropical Medicine Mahidol University Bangkok Thailand

**Keywords:** health care information system, DEPIC, mobile technology, maternal and child health, mHealth, vaccine record, electronic data capture

## Abstract

**Background:**

Entering data onto paper-based forms, then digitizing them, is a traditional data-management method that might result in poor data quality, especially when the secondary data are incomplete, illegible, or missing. Transcription errors from source documents to case report forms (CRFs) are common, and subsequently the errors pass from the CRFs to the electronic database.

**Objective:**

This study aimed to demonstrate the usefulness and to evaluate the effectiveness of mobile phone camera applications in capturing health-related data, aiming for data quality and completeness as compared to current routine practices exercised by government officials.

**Methods:**

In this study, the concept of “data entry via phone image capture” (DEPIC) was introduced and developed to capture data directly from source documents. This case study was based on immunization history data recorded in a mother and child health (MCH) logbook. The MCH logbooks (kept by parents) were updated whenever parents brought their children to health care facilities for immunization. Traditionally, health providers are supposed to key in duplicate information of the immunization history of each child; both on the MCH logbook, which is returned to the parents, and on the individual immunization history card, which is kept at the health care unit to be subsequently entered into the electronic health care information system (HCIS). In this study, DEPIC utilized the photographic functionality of mobile phones to capture images of all immunization-history records on logbook pages and to transcribe these records directly into the database using a data-entry screen corresponding to logbook data records. DEPIC data were then compared with HCIS data-points for quality, completeness, and consistency.

**Results:**

As a proof-of-concept, DEPIC captured immunization history records of 363 ethnic children living in remote areas from their MCH logbooks. Comparison of the 2 databases, DEPIC versus HCIS, revealed differences in the percentage of completeness and consistency of immunization history records. Comparing the records of each logbook in the DEPIC and HCIS databases, 17.3% (63/363) of children had complete immunization history records in the DEPIC database, but no complete records were reported in the HCIS database. Regarding the individual’s actual vaccination dates, comparison of records taken from MCH logbook and those in the HCIS found that 24.2% (88/363) of the children’s records were absolutely inconsistent. In addition, statistics derived from the DEPIC records showed a higher immunization coverage and much more compliance to immunization schedule by age group when compared to records derived from the HCIS database.

**Conclusions:**

DEPIC, or the concept of collecting data via image capture directly from their primary sources, has proven to be a useful data collection method in terms of completeness and consistency. In this study, DEPIC was implemented in data collection of a single survey. The DEPIC concept, however, can be easily applied in other types of survey research, for example, collecting data on changes or trends based on image evidence over time. With its image evidence and audit trail features, DEPIC has the potential for being used even in clinical studies since it could generate improved data integrity and more reliable statistics for use in both health care and research settings.

## Introduction

Paper-based case report forms (CRFs) have long been used as the standard method to collect data in research studies and health care services [1]. Both primary and secondary data are collected in many public health surveys using paper-based CRFs. Once data are collected, they should be accurately entered, coded into an electronic form, and subsequently converted into many forms for further analysis [2]. However, this approach presents many problems due to frequent errors and high storage costs when performing data collection and double data entry [3]. Moreover, there are many problems that can arise after data collection—especially when collecting secondary data—including lost forms, incompletely filled forms, and poor handwriting of data collectors. There are multiple potential sources of error that can occur when performing manual data collection, particularly if data collection involves multiple data collectors across multiple health care units, or even if data collection is done within 1 unit [4-5]. Mobile phones offer an attractive possibility to address these problems in terms of their accessibility, effectiveness, and quality of data that includes data completeness and validation.

It is suggested in literature that several mobile phones features have created opportunities for data collection, and that these features could also improve data quality [3,6-9]. Mobile phone cameras have been used as an alternative method for health care data collection in recent years, although mobile phone cameras are still mostly used in capturing clinically relevant images for rapid diagnosis [10]. For example, the use of mobile phones by medical doctors to view medical image data such as neurosurgery and dermatology for rapid and convenient diagnosis has been reported [9,11,12]. Other examples include the use of mobile phone imaging in microscopic diagnosis of soil-transmitted helminthic infections and diagnosis of sputum slides for TB. [10,13-15]. Although mobile phone cameras are useful in health care data collection, usage should be carefully planned due to a higher equipment cost, lack of ability to verify miscoded data against paper records once data is entered, and the varying quality of images taken with different mobile phones [10,16].

In Thailand, mobile phones have been used as data collection tools in the health care system. A project supported by Google Thailand developed and employed mobile applications for health data collection in the Northern provinces. The data collected via mobile phones were compared to paper CRFs flowing directly to the hospital electronic database and the health care providers, and policy makers could see all details of individual health data of the entire district [17]. Another study conducted in northern Thailand showed that the customized-language voice surveys for textual data, together with capturing image data on mobile phones, could be successfully used to collect data among ethnic populations who speak different languages [8]. Moreover, 1 project in Thailand supported by Microsoft Research showed the effectiveness of using mobile technology in routine health care services that focused on reminding an individual of scheduled visits for antenatal care and Expanded Programme on Immunization (EPI) services [18]. Similarly, another project in Thailand focused on malaria case management by implementing a module on mobile phones to monitor and follow malaria cases, including patient treatment [19]. Various studies revealed that mobile technology is the fastest growing sector in the communications industry, especially in low-income countries [20]. Thus far in the context of poor resource settings for large-scale public health surveys, the availability and affordability of mobile phones and wireless networks create a possible alternative mechanism for data collection that might replace traditional paper-based methods.

**Figure 1:**
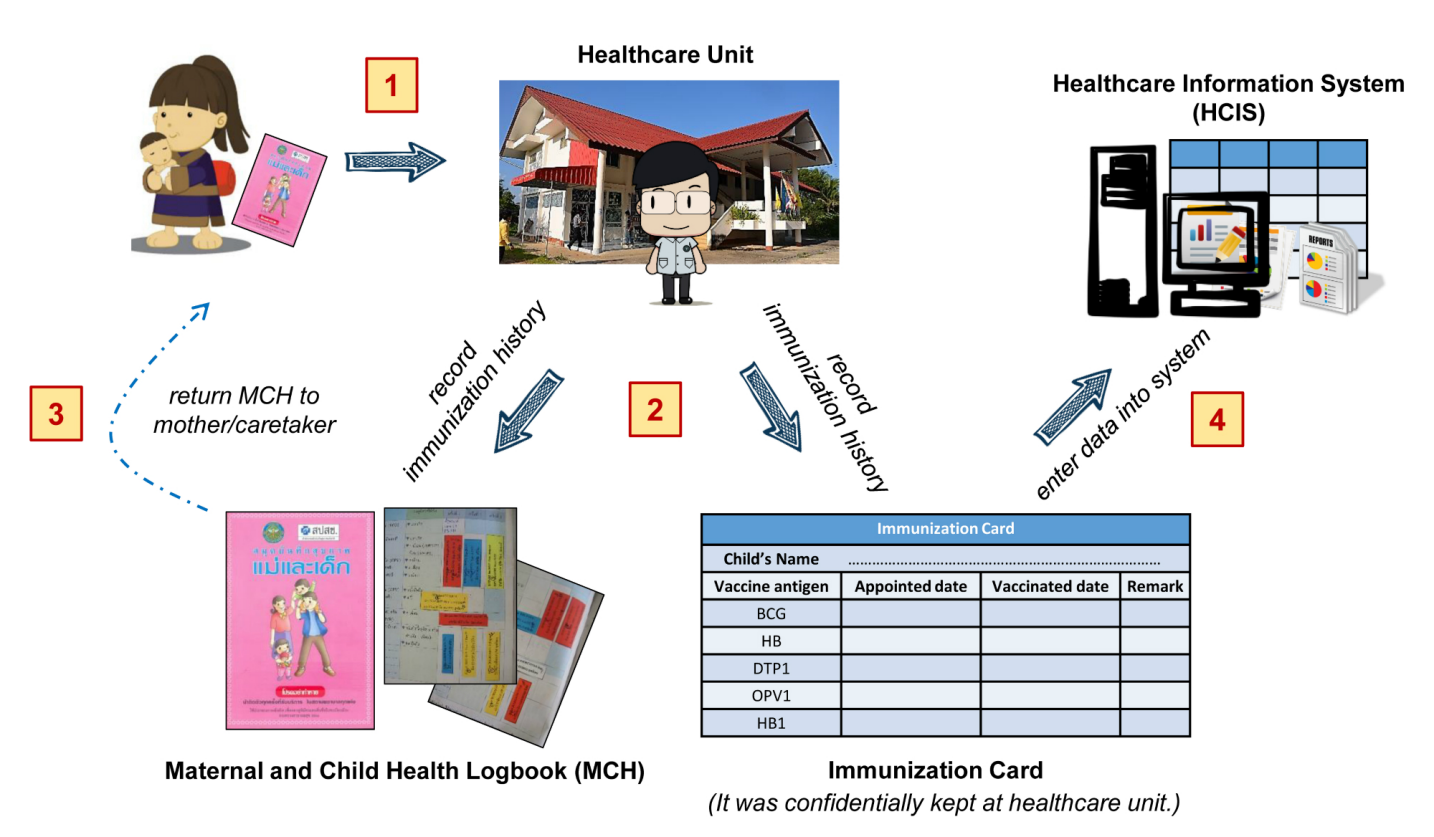
Routine immunization service at health care unit.

The routine work on immunization services at a primary health care unit consists of 4 steps as presented in Figure 1. As an enforced routine practice of the Thailand Ministry of Public Health, a MCH logbook is given and owned by 1 mother/caretaker and every health care unit that provides the service asks for the MCH logbook and records the child’s immunization history into the logbook every time the mother/caretaker brings a child for immunization. As shown in Figure 1 as step 1, on a vaccination day, the mother/caretaker presents the logbook to the health care provider at the primary health care unit. In step 2, the health care provider gives the immunization(s) as per schedule then separately records the vaccine(s) administered, actual date of immunization, and the date for the next appointment on 2 documents, the MCH logbook and the individual immunization history card. In step 3, the MCH logbook is returned to the mother/caretaker while the individual immunization history card is kept at the primary health care unit. In step 4, the record on the immunization history card is entered into the national health care information system (HCIS) by health care providers, which is when data problems usually occur. There is a time gap between when data are entered on the immunization history card to when data are entered into the HCIS; data entry cannot be done in real time due to the workload of health care providers on the vaccination day. And since it has been generally recognized that data in the HCIS are incomplete or missing, any reports/statistics about immunization generated from the HCIS database will be unreliable.

It would be a great challenge to change the 4-step routine practice by having health care providers enter the data into the HCIS at the same time they provide the services and discard the use of MCH logbooks and individual immunization history cards. In Thailand, the problems of the health sector at district level are limited human resources and inadequate infrastructure. It is difficult to carry out data entry while providing services to a large number of patients. The MCH logbooks usually have an almost-complete immunization history of each child, and the mother/caretaker who owns it often use it as an immunization schedule reminder. The mother/caretaker is required to bring the MCH logbook to every scheduled immunization, and health care providers usually rely on the information in the logbook more than that in the HCIS. Collecting secondary data for further analysis using source documents or logbooks can be another challenge due to difficulties in reading, extracting, and transcribing such information, especially if the data are collected by those who are not familiar with all information content and context.

This study aimed to demonstrate the use and evaluate the effectiveness of the camera function on mobile phones combined with online connectivity as a tool for health data collection. The effectiveness of data image capture feature was assessed through data quality in terms of completeness and consistency of the records by comparing the captured images with the data on the national HCIS database. In addition, the impact of data quality was confirmed through comparisons of some statistics generated from the 2 data sources.

## Methods

### Study Sites and Study Participants

**Figure 2:**
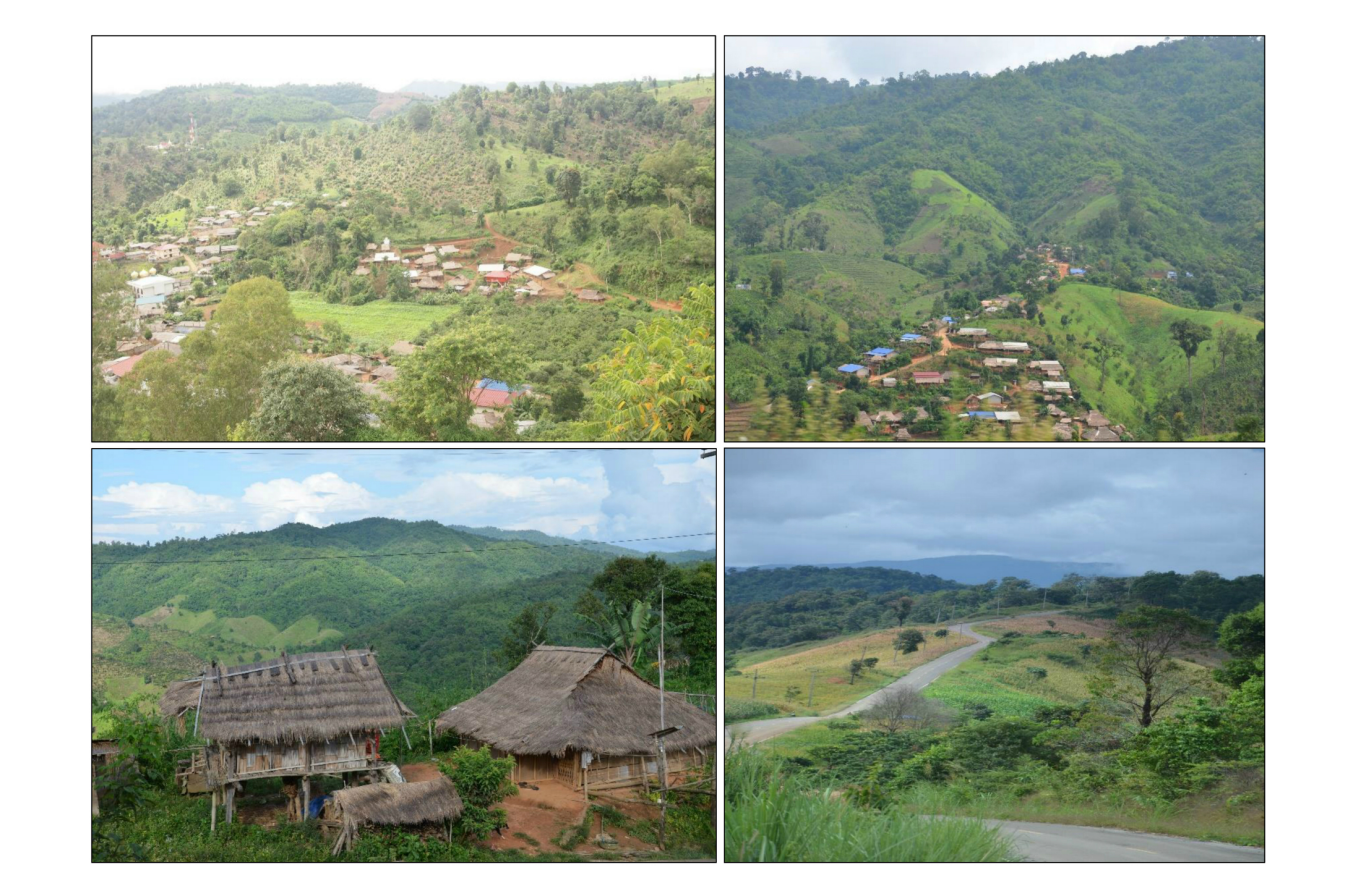
Study sites were located on the highlands of northern Thailand.

This cross-sectional study was conducted in 8 villages in the Wawee Subdistrict of the Mae Suai District, which is in the Chiang Rai Province of northern Thailand, during May through August 2013. The majority of people in these areas are from ethnic groups, including Karen, Lahu, Lisu, Hmong, Mien, Yunnan Chinese, and Akha; some of which have no writing system. Village health volunteers (VHVs) in these villages were recruited as data collectors. They were trained to collect data from MCH logbooks using a mobile phone camera application equipped and were assigned to make home visits. Images of immunization history records were then captured from the MCH logbook of each hill tribe child. Data to be used for an analysis are those recorded on MCH logbooks from the child’s first immunization until the end of April 2013. Since there were 790 children under 6 years of age in these 8 villages, the 363 mothers possessing the children’s immunization logbooks were randomly selected using simple random sampling technique. Figure 2 shows some of the study sites where these minority groups are located in the highlands.

### Implementation of DEPIC

DEPIC stands for “data entry via phone image capture.” DEPIC was developed as part of the smartphone survey project initiated by the Center of Excellence for Biomedical and Public Health Informatics (BIOPHICS) at the Faculty of Tropical Medicine, Mahidol University, Thailand. The smartphone survey application ran on Android SDK, and was built using Eclipse open-source software. DEPIC was an enhancement of the survey tool performing 1 of the 3 main features of the smartphone survey application. The details of the other 2 features of the smartphone survey, including drop-down menu choice and voiced-questioning in selectable ethnic languages, are discussed elsewhere [8]. This smartphone survey tool application was successfully developed and tested in the previous study in northern Thailand among different ethnic minority groups. The previous study was conducted to assess data quality in terms of data completeness and time consumed in collecting the information in comparison with traditional data collection methods (eg, paper-based questionnaire). Besides data quality, the participants’ satisfaction with the smartphone customized-language voice-based questionnaire in terms of perceived ease of use and perceived usefulness was assessed [8]. The particular purpose of the DEPIC application was to reduce the workload and form-filling mistakes by data collectors in the field. The data image capture functionality on mobile phone was employed to make it faster and easier to collect secondary data, with no need to extract data from the source document, enter the data onto CRFs, and reenter the data again into the electronic database. DEPIC can be used either online and automatically synchronized with a central database or offline and synchronized with a central database when telephone signals or wireless networks are available.

**Figure 3:**
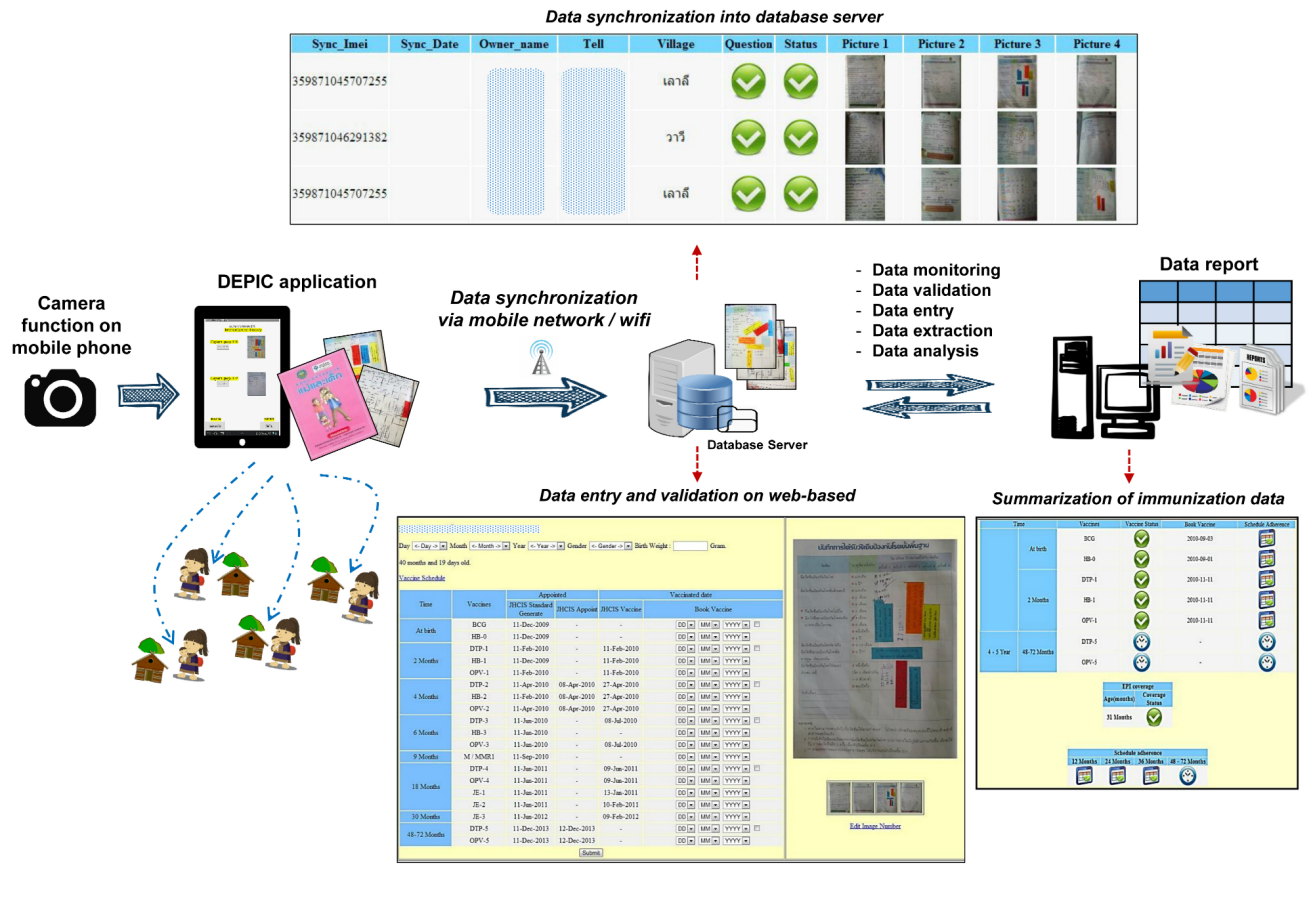
DEPIC conceptual framework.

In this study, DEPIC was used to collect immunization history records (eg, prescheduled date and actual vaccination date) from mother and child health (MCH) logbooks. A conceptual framework of the DEPIC feature is shown in Figure 3. The image-taking was designed to decrease the workloads of VHVs while interviewing hill tribe mothers/caretakers so that the health workers did not need to manually extract data from the logbook and transcribe the data onto paper CRFs. While working in the field, VHVs who performed routine monthly home visits simply asked for the MCH logbook from the mothers, captured the data image of the immunization history pages via the DEPIC application, and saved the image automatically in the mobile phone. Each picture was then synchronized to the central database, where an electronic Web-based form was created according to the transmitted picture. If the picture was not clear, the VHVs repeated the picture-capturing process until a suitable image was captured. DEPIC mapped picture images with a data entry screen for each child’s logbook. Data were then manually entered by the data management team and submitted to the study investigator. At this phase of DEPIC development, there are no features of automatic character (ie, text) recognition and no double data entry from the image; these are in the planning phase. The purpose of this study is to capture presumably complete data from logbooks to compare against the data in the health care unit’s HCIS database.

### Data Linkage for Comparison

To demonstrate the use of mobile technologies in data capturing, the immunization history data from 2 databases were compared. Data collected via DEPIC were compared with data points in the standard HCIS database. Data in the DEPIC database represent complete immunization history data that were actually recorded in a MCH logbook during the immunization process by the health care providers on the scheduled immunization dates. The data extracted from HCIS database represent data entered ad hoc by health care providers from duplicate information of the logbook data on individual immunization history cards after the immunization process. Data between the 2 databases, DEPIC and HCIS, were linked by each child’s hospital number to extract both the appointment date and the actual vaccination date of each child. The matching of the data was done using Excel and then transported to a statistical package for further analysis. The data fields in the MCH logbooks are always more complete than those in the HCIS; there were no data fields that were found in the HCIS but not in the logbooks.

### Data Definitions and Analysis

For the purposes of this study, completeness was defined as all records being entered into the database, with no missing or incomplete data. Consistency was defined as the absence of typographical and transcription errors which may lead to differences in the immunization history data between the 2 databases. The comparisons of completeness and consistency of the data in the 2 databases were performed on immunization history records in 2 aspects: percentage of completeness of the number of immunization history records and consistency of the actual vaccination date(s) reported in each record. The completeness of the number of records for each MCH logbook was determined by the number of immunization records that were not entered into the HCIS but were captured and presented in DEPIC. The consistency of the individual actual vaccination date was determined by the number of records in each MCH logbook that such dates were matched between the 2 databases, DEPIC and HCIS.

In order to assess the impact of completeness and consistency of the data in the 2 databases, derived statistics on immunization coverage and compliance to immunization schedule status were calculated and compared. Immunization coverage status was displayed in individual summary statistic, as well as the immunization schedule compliance status of the district. The status of immunization coverage was categorized into 2 groups: “complete immunization” and “incomplete immunization.” The complete immunization status was applied if a child had fully received the correct number of doses of all vaccines following the immunization schedule by child’s age, while the incomplete immunization status was applied if a child had missed at least 1 dose of all vaccines. Regarding the compliance to the immunization schedule, the term “compliance” in this study referred to when the child completely received the correct number of doses of each vaccine according to time (ie, the child’s age) and sequence of vaccines, as stated in the Thailand immunization schedule guideline [21,22]. The compliance to immunization schedule status was classified into 3 levels: “on time,” “out of schedule,” and “pending schedule.” The on time status applied when the child had fully received a number of doses of all vaccines according to the time sequence in the guideline. The out of schedule status applied when a child had fully received a number of doses of all vaccines, but at least 1 vaccine did not follow the time sequence according to the guideline. The pending schedule status meant that the child was not required to be immunized with the particular vaccines at the analysis time.

### Ethical Considerations

This study was a part of the project “Assessment of Immunization Status of Hill Tribe Children Using Multilingual Audio Visual Mobile Technology.” The project was reviewed and approved by the Ethics Committee of the Faculty of Tropical Medicine, Mahidol University. This study involved vulnerable research participants belonging to ethnic groups in the Chiang Rai Province of Thailand. All participants were informed about all details regarding the study and asked to sign an informed consent form before participating. The document was translated by VHVs into the participants’ dialect or language.

There was no identification of first or family name of the respondents on the CRFs. The individual information was kept completely confidential during data collection and analysis. The respondents were able to stop participating at any time and did not need to give a reason for the withdrawal of their consent.

### Data Security and Storage

All captured pictures of immunization history records were kept confidentially on mobile phones designated to each VHV, who was responsible for his/her own catchment villages. In this study, all of these pictures were synchronized and transferred for analysis at the central database at BIOPHICS with a secured system to ensure limited accessibility and scheduled backups. Data entry and analysis was done by the investigators on a designated computer that was locked using a secured password.

## Results

### Use of DEPIC for Data Capture in the Field

The DEPIC tool was developed for use in the field with minor effort, as camera functionality is normally available on most cell phones, mobile phones, and tablets. Android platforms also enable the development of customized camera applications. The application was found to be easy to use and required few hours of training for the VHVs, who comprised were ethnic people living in the remote areas and acted as the data collectors in the project. The VHVs reported that they felt capable of using the application in collecting the secondary data of the MCH logbook. The VHVs agreed that they could take pictures and submit them to the data center with minimum effort. Of 726 page-pictures from 363 records, only 64 pages of data images (8.82%) had to be re-submitted. Based on the observations at the study sites, the health care personnel who worked with the HCIS database suggested that an application like DEPIC could increase data quality within the national database system, as well as the efficiency of survey data collection. With the current version of DEPIC, the submitted images were automatically transferred to the central data center whenever the telephone signal was available; however, they were not automatically read. The data entry people had to key in the data from the image into a pop-up data screen that matched the images received. The clear images facilitated the data entry process. The data entry people were satisfied with the task assigned to them. The information in the MCH logbook was comprised mostly of check-boxes and data fields with immunization information filled in using preprinted stickers prepared by the health care unit. In the case that the immunization was not performed at the participant’s primary health care unit, such information was handwritten.

### Differences of Immunization History Records Between HCIS and DEPIC

During the study period, 363 hill-tribe mothers/caretakers from 8 villages were randomly selected for participation in the project; they were requested to submit the MCH logbooks to VHVs for capturing the immunization history records using DEPIC. DEPIC and HCIS records were matched for all 363 mothers’ and children’s identifications. Considering the images taken from MCH logbooks via DEPIC as complete, completeness and consistency of immunization history records were assessed by comparing the 2 databases, DEPIC and HCIS, as presented in Table 1. In terms of completeness of immunization history records, 17.3% children (63/363) had totally different immunization history records when looking into DEPIC and HCIS; complete immunization history records were found in DEPIC, but none in HCIS. Regarding the consistency of actual vaccination dates, the information taken from MCH logbooks was compared to data in HCIS and it was found that 31.1% (113/363) of the records’ dates in HCIS matched dates in DEPIC 51%-70% of the time, 28.4% (103/363) of records matched 50% or less, and for 24.2% (88/363) no dates matched. It should be noted that no records matched 100%.

**Table 1:** Difference of immunization history records between DEPIC and HCIS.

Variables		Number (N=363)	Percentage (%)
**Completeness of immunization history records (DEPIC vs HCIS)^a^**			
	≤ 50% difference	238	65.6
	51%-80% difference	49	13.5
	81%-99% difference	13	3.6
	100% difference	63	17.3
**Consistency of actual vaccination dates (DEPIC vs HCIS)^b^**			
	100% unmatched	88	24.2
	≤ 50% matched	103	28.4
	51%-70% matched	113	31.1
	> 70% matched	59	16.3

^a^The percentage of completeness was determined as number of records that were not entered in HCIS but presented in DEPIC for each MCH logbook.

^b^The percentage of individual actual vaccination date consistency was determined as number of records that were matched between DEPIC and HCIS for each MCH logbook.

### Differences of Immunization Coverage Status Between DEPIC and HCIS

One of the purposes of collecting the immunization records was to assess immunization coverage among the targeted populations. In this study, the focus was on immunization coverage within the first year of age. Individual records from both DEPIC and HCIS were calculated in 2 dimensions: immunization coverage status (both overall and by each vaccine antigen) and compliance to immunization schedule status. Differences of calculated immunization outcomes from the 2 databases are presented in Table 2. The number of individuals who had complete immunization according to DEPIC records was higher than that derived from HCIS records (ie, 79.1% (287/363) vs 0.3% (1/363)). For immunization coverage status by each vaccine antigen, records stored in DEPIC revealed that all children in the study received the BCG vaccine, and the immunization rates of the other vaccines were more than 90%. In contrast, the records in HCIS indicated that immunization rates of different vaccines varied from 1% to 74%. That is, status of complete immunization in each vaccine antigen was shown to be much higher with DEPIC than HCIS.

**Figure 4:**
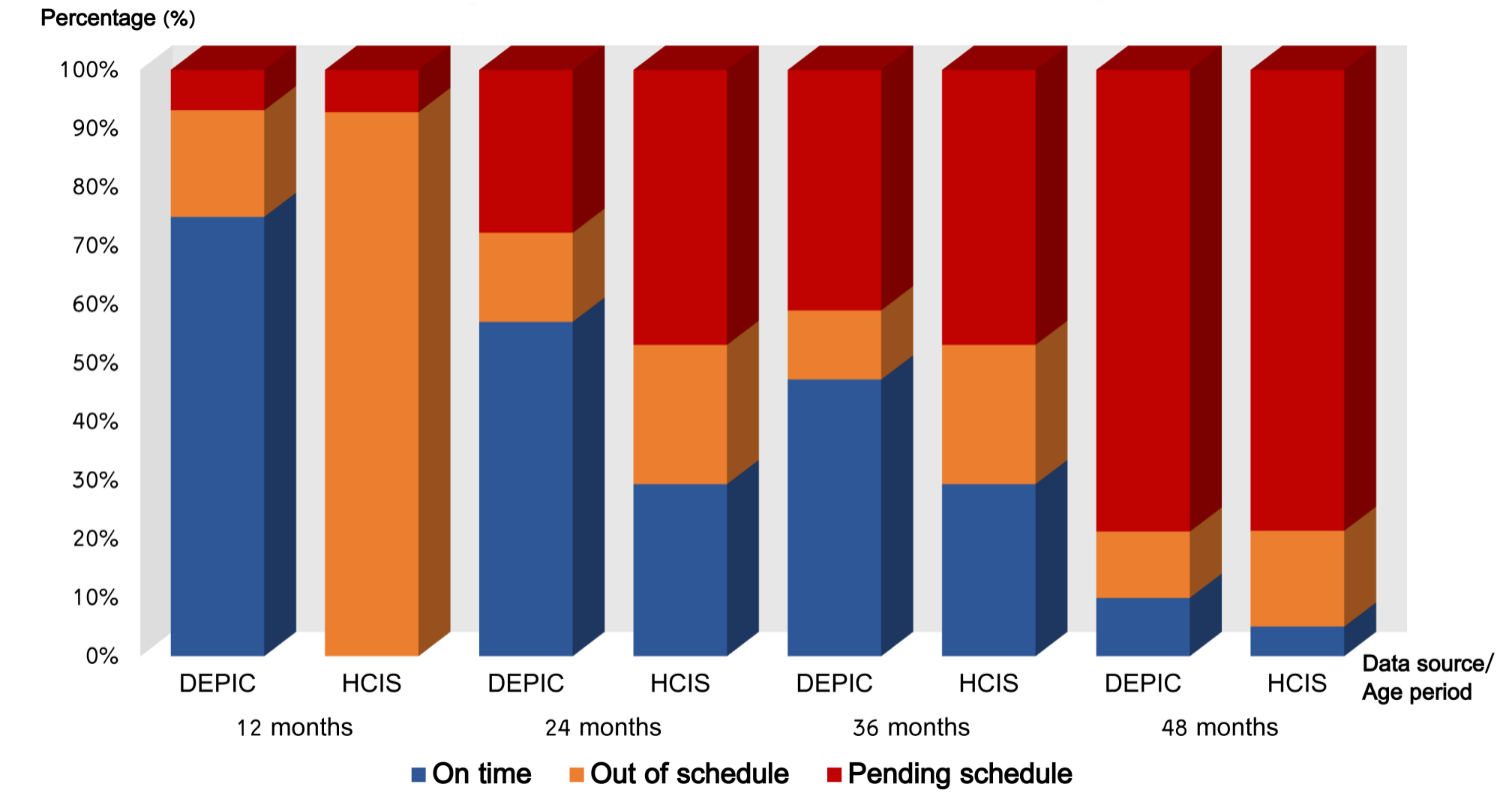
Status of compliance to immunization schedule by data source.

Status of compliance to immunization schedule by age group revealed different outcomes, as shown in Figure 4. When the calculation was based on the records in DEPIC, more children were immunized according to the scheduled time sequence. According to the DEPIC records, 74.9% (272/363) of children received vaccines on time during their first year of age (ie, 12 months), while HCIS records showed the result of 0% (ie, no children received the vaccines on time). The same patterns were found for subsequent age groups.

**Table 2:** Data analysis of immunization outcomes between DEPIC and HCIS.

Immunization Coverage Status		DEPIC		HCIS	
		Number (N=363)	Percentage^a^ (%)	Number (N=363)	Percentage^a^ (%)
**Overall status**					
	Complete immunization	287	79.1	1	0.3
	Incomplete immunization	76	20.9	362	99.7
**Status by vaccine antigen**					
	BCG	363	100.0	73	20.1
	DTP1	362	99.7	201	55.4
	HB1	362	99.7	212	58.4
	OPV1	362	99.7	268	73.8
	DTP2	359	98.9	254	70.0
	HB2	359	98.9	253	69.7
	OPV2	359	98.9	249	68.6
	DTP3	354	97.5	231	63.6
	HB3	354	97.5	5	1.4
	OPV3	354	97.5	226	62.3
	M/MMR1	338	93.1	23	6.3

^a^The percentage was calculated from number of children with an immunization schedule.

## Discussion

### Principal Results

Differences in the completeness of immunization history records and consistency of individual actual vaccination dates for each record in the MCH logbooks between 2 databases, DEPIC and HCIS, reflect the problematic situation of data entry of immunization records into the national database system in Thailand. This study finds that should the data recorded in MCH logbooks be simultaneously entered directly into the electronic database—rather than being recorded on the individual immunization history cards before being entered into the electronic database—there will be more complete and accurate data in the national HCIS database. The simple explanation for incomplete and missing information in the HCIS database is that data entry into HCIS is usually delayed due to case-management workloads on prescheduled immunization days. Moreover, the individual immunization history cards used as source documents for HCIS data entry are often incomplete. It doubles the work for health care providers to collect data during the vaccination day on both data sources: the MCH logbook, which is kept by the mother/caretaker, and the individual immunization history card, which is kept by the health care unit for ad hoc entry into HCIS. Health care providers also might be more likely to miss recording the data on the card but complete the MCH logbook since the logbook record is fully enforced by the Thailand Ministry of Public Health and is used by the mother/caretaker as a reminder for the next scheduled immunization. It is thus assured that the child’s complete immunization history will be recorded and can be found in the MCH logbook.

The incompleteness of immunization information in the HCIS database as compared to data captured via DEPIC is also reflected in the statistics on vaccine coverage and compliance to the immunization schedule for children. Using statistics calculated from information in the HCIS alone, it appears that Thailand has lower immunization coverage and compliance according to the national guideline. But when compared with statistics calculated from the DEPIC records, it appears to be quite the opposite with study participants showing high immunization coverage and compliance rates. Therefore, comparing data quality on immunization history among children in remote areas as an example, we suggest that DEPIC could be used to collect data quite effectively.

The DEPIC was implemented in the field with minimum requirements. Data images were captured and automatically submitted to the data center when there was telephone signal. In this study in a remote area, data images were collected and submitted by local VHVs who had limited education levels. They expressed that it was not a burden to collect DEPIC data while performing their routine home visits. They collected data for health care providers easily, and not much effort was required. At the local health centers in the study locations, health care personnel indicated that DEPIC would help them cut down the workload if it was redesigned as a mobile technology tool for use by the health care personnel at the local center for data capture. This is an issue that needs further planning and collaboration in order to lessen the workloads of health care providers by using DEPIC for data image capture onto HCIS, rather than the current system of entering data twice, first into an MCH logbook and then onto an individual immunization history card for HCIS.

The development of DEPIC was based on the idea of creating a data collection tool for capturing secondary data in survey research. DEPIC has shown its potential use in collecting secondary data as a direct image when it is difficult for data collectors to collect the secondary data on paper-based CRF. This is due to the difficulties in reading, recalling, and writing such information. This method is quite effective as it can capture data directly from source documents (eg, MCH logbook) and, thus, there is no need to perform data extraction and/or source data verification (ie, cross-checking between source documents and CRFs). As also demonstrated in other studies using mobile technology in remote areas [23,24], it simplifies the process of survey data collection in remote areas where study participants speak other languages. As found in many previous studies that used paper-based methods in collecting secondary data, such methods appear to have more transcription errors and missing data [25-32]. Transcription errors could occur at 2 stages—from source document to paper-based CRF, and then from CRF to database. The DEPIC application acts as a direct electronic CRF, thus halving the sources of error in the data-capture process. The implementation of DEPIC in this study suggests that it helped reduce the time consumed for data collection. Other studies have also reported decreased time spent in data collection using mobile technology [16,30,33].

In this study, data captured via DEPIC and entered into the HCIS database for further analysis has shown to improve the accuracy and completeness of data. One of the advantages of using DEPIC is that it provides evidence-based data via photographic images from the original source document. As shown in this study, DEPIC can capture all relevant information regarding the immunization history of children. Data stored in the DEPIC database were more complete, with supporting electronic evidence as an audit trail, and so could provide more reliable statistical analyses when needed. It should be noted, however, that errors or incomplete data could occur even with DEPIC due to unclear pictures—even if VHVs usually use mobile phones every day—and this might require retaking of the data image. It is important that the data collectors who use the device receive appropriate training for proficiency in camera use when a new data collection tool/application is introduced or if new survey content is planned.

### Limitations of Current Version of DEPIC

The results of this study confirmed the potential implications of using camera applications on mobile devices in various ways to provide health care services, as is shown in previous studies [1,9-15,34]. In this study, DEPIC was developed and used for a simple descriptive survey, particularly to collect immunization history data from MCH logbooks or immunization cards. We recognize that in the comparison of data quality in this example of immunization history recording we assumed that the MCH logbooks are the complete and correct data source and, relying on these alone, we suggest that data quality captured by DEPIC is better than data in the national data source. It should be noted that we did not claim that the DEPIC provides more accurate data, but rather focused on completeness and consistency of data between the 2 data sources. There are several potential sources of inaccuracies; for examples, in either data sources, vaccines may be misrecorded, may be replaced with missing/incorrect data, or may be written as given when immunization did not actually happen. The results in this study simply demonstrate that (1) DEPIC could be used as a data collection tool to capture complete data with data images rather than the traditional paper-based data collection-and-entry method that results incomplete data; and (2) that DEPIC requires less effort and time to collect secondary data by cutting down the typical steps from extracting data from source documents to paper-based CRFs and then from CRFs to electronic databases.

In this study, the DEPIC was implemented as an example for use in data collection of a single survey. The DEPIC concept, however, can be easily applied as a data collection tool in other types of survey research; for example, collecting data on changes or trends based on image evidence over time. With its image evidence and audit trail features, DEPIC has potential applications for use even in clinical studies. With the purpose to prove the concept of using DEPIC for secondary data, the current application does not yet allow for double data entry to cross-check data validity. To comply with best data-entry practices in clinical studies, the next version of DEPIC should incorporate a double-entry function from image capture.

### Conclusions

DEPIC, or the concept of collecting image data as a primary source, has proven be a useful data collection method. It was found to be superior to paper-based methods in regard to the consistency and completeness of data. As a case study of using DEPIC to capture immunization history data among minority populations in remote areas, this study shows that the concept can be applied in a limited-resource environment. With traceable evidence-based images and better data quality, the DEPIC concept also has a potential to generate improved data integrity and more reliable statistics for use in public health and research settings.

## Abbreviations

BIOPHICS: Center of Excellence for Biomedical and Public Health Informatics
CRF: case report form
DEPIC: data entry via phone image capture
HCIS: health care information system
MCH: mother and child health logbook
VHVs: village health volunteers
